# Testing the translocation hypothesis and Haldane’s rule in *Rumex hastatulus*

**DOI:** 10.1007/s00709-018-1295-0

**Published:** 2018-08-03

**Authors:** Magdalena Kasjaniuk, Aleksandra Grabowska-Joachimiak, Andrzej J. Joachimiak

**Affiliations:** 1 Department of Plant Breeding and Seed Science, University of Agriculture in Kraków, Łobzowska 24, 31-140 Kraków, Poland; 2 Department of Plant Cytology and Embryology, Institute of Botany, Jagiellonian University, Gronostajowa 9, 30-387 Kraków, Poland

**Keywords:** *Rumex hastatulus*, Sex chromosomes, Hybrids, Haldane’s rule, Meiosis, Fertility, Sex ratio

## Abstract

The translocation hypothesis regarding the origin of the XX/XY1Y2 sex chromosome system was tested with reference to the F1 hybrids between two chromosomal races of *Rumex hastatulus.* The hybrids derived from reciprocal crossing between the Texas (T) race and the North Carolina (NC) race were investigated for the first time with respect to the meiotic chromosome configuration in the pollen mother cells, pollen viability, and sex ratio. A sex chromosome trivalent in the NC × T males and two sex chromosome bivalents in the T × NC males were detected. The observed conjugation patterns confirmed the autosomal origin of the extra chromosome segments occurring in the North Carolina neo-sex chromosomes. Decreased pollen viability was found in the T × NC hybrid in contrast to the NC × T hybrid and the parental forms. Moreover, only in the T × NC hybrid sex ratio was significantly female-biased (1:1.72). Thus, Haldane’s rule for both male fertility and male rarity was shown in this hybrid. According to the authors’ knowledge, *R. hastatulus* is just the second plant with sex chromosomes in which Haldane’s rule was evidenced.

## Introduction

Sex chromosomes in flowering plants are relatively young compared to mammalian ones; thus, they provide an excellent opportunity to study the early stages of differentiation of X/Y chromosomes and sex chromosome systems (Charlesworth 2002, 2015; Ming et al. 2011). Generally, it is believed that the mechanisms of sex chromosome evolution are similar in plants and animals, but issues concerning plant sex chromosomes such as the rate and extent of addition and attrition of genetic material, localization, structure and function of sex-determining regions, and the occurrence (or not) of dosage compensation mechanisms have not been well recognized yet (Vyskot and Hobza 2004; Charlesworth et al. 2005; Charlesworth 2008; Ming et al. 2011; Bergero et al. 2015; Beaudry et al. 2017; Crowson et al. 2017; Muyle et al. 2017). Also, little is known about the cytogenetic and molecular mechanisms underlying the formation of neo-sex chromosomes in plants (Navajas-Pérez et al. 2005; Charlesworth and Mank 2010).

*R. hastatulus* is an annual dioecious North American species with two races (Texas and North Carolina) differing in both the chromosome number and sex chromosome system (Smith 1963). The Texas (T) race possesses the simple sex chromosome system (XX/XY) and equal chromosome number in males and females (2*n* = 10). The North Carolina (NC) race shows the multiple (polymorphic) sex chromosome system (XX/XY1Y2) which makes the sexes differ in the chromosome number (2*n* = 8 in females and 2*n* = 9 in males). According to Smith (1963), in the NC race, sex is generally determined by X-to-autosome balance (X/A), but Y chromosomes are not neutral in gender determination because they contain genetic material necessary for expression of maleness (this system is intermediate between X/A balance observed in *Rumex acetosa* and the system with active Y observed in *Silene latifolia*). Such an intermediate sex-determining system was also confirmed in the T race by Bartkowiak (1971).

**Fig. 1:**
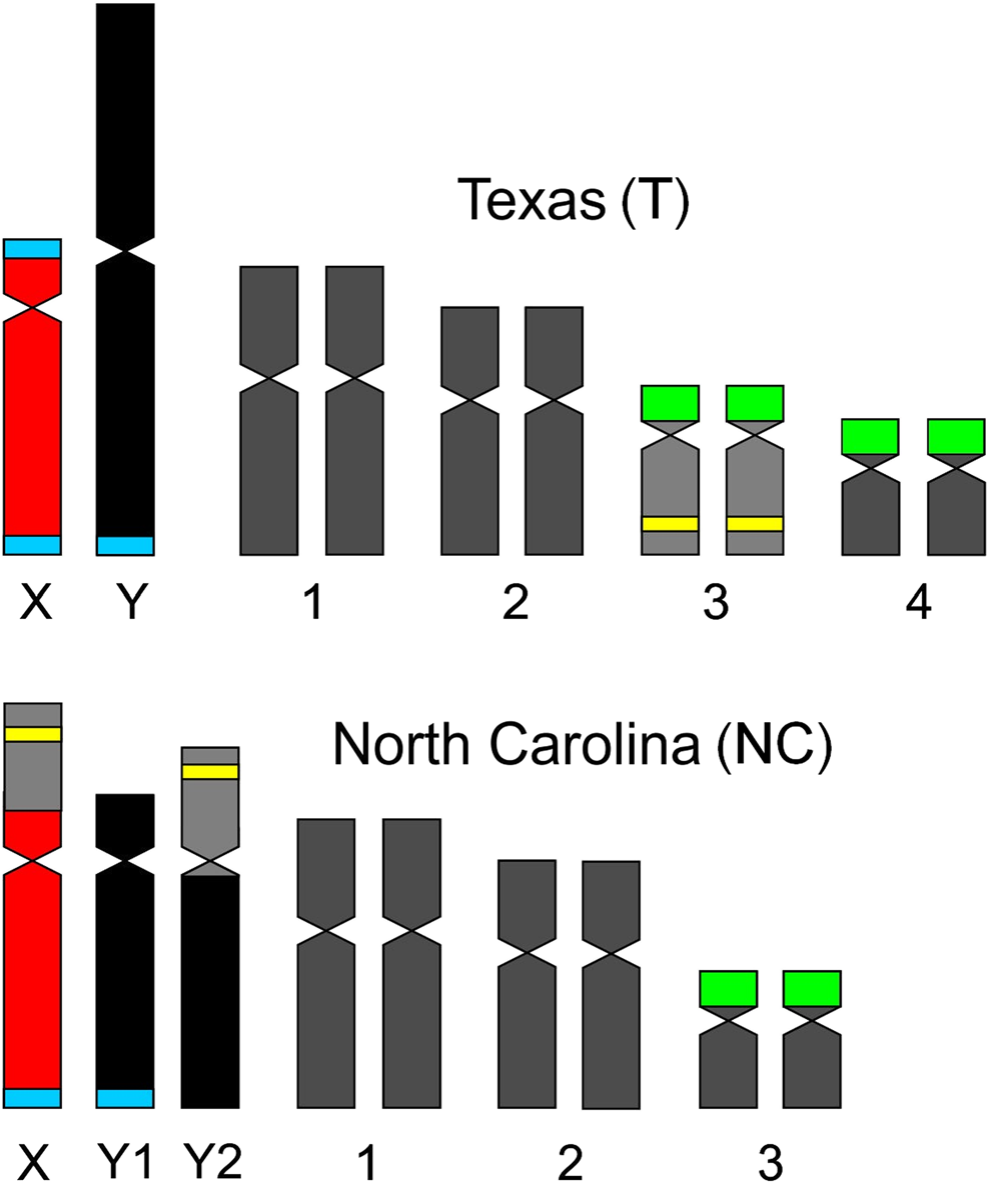
Chromosome complements of two *R. hastatulus* races. 35S rDNA sites (*green*), 5S rDNA sites (*yellow*), ancestral pseudoautosomal segments (*blue*)

Smith (1964) suggested that the NC karyotype originated from the T karyotype through autosome-sex chromosome translocations resulting in the dysploid reduction and emergence of neo-sex chromosomes (translocation hypothesis). C-banding/DAPI method, FISH with rDNA probes, and flow cytometry confirmed this scenario and showed the involvement of the small third autosome pair originally equipped with 5S and 35S rDNA in this event (Grabowska-Joachimiak et al. 2015). The karyotype underwent further changes consisting in elimination of 35S rDNA from translocated autosomes, fission of the primeval Texas Y chromosome, and genome downsizing. As a result, the neo-sex chromosomes were formed: neo-X (X_NC_; the original X + autosome), Y1 (centric fragment of the original Y), and Y2 (fragment of the original Y + autosome) (Fig. 1). The pseudoautosomal regions (located in X_NC_ and Y1) inherited from the ancestor and autosome pair translocated on X_NC_ and Y2 chromosomes ensured formation of the regular sex trivalent (Y1-X_NC_-Y2). Thanks to alternate disjunction of the neo-X from the two Ys, two A + X_NC_ and two A + Y_1_Y_2_ microspores in each tetrad were produced, and the modified sex chromosome system became stabilized.

Although the origin of the polymorphic sex chromosome system in *R. hastatulus* seems to be well documented based on karyotype analysis and observation of sex trivalent in the NC males, its final confirmation requires proving that the neo-sex chromosomes of this race are able to conjugate with the ancestral third autosome of the Texas race.

The emergence of the simple (XX/XY) sex chromosome system occurring in the T race of *R. hastatulus* can be related to the appearance of dioecy in *Rumex*, estimated between 15 and 16 million years ago (Navajas-Pérez et al. 2005). On the other hand, the split between the T race and the NC race occurred very recently, around 600,000 years ago (Navajas-Pérez 2012). To our knowledge, the sex chromosome system of the NC race is the youngest among all plants possessing heteromorphic sex chromosomes studied so far. Two different sex chromosome systems within a single species and a very young age of *R. hastatulus* neo-sex chromosomes provide a unique opportunity to study the patterns of sex chromosome evolution in plants.

The lack of recombination between X and Y chromosomes leads, among others, to accumulation of repetitive sequences and heterochromatinization of Y chromosome (Matsunaga 2009). This is not the rule in all plants with heteromorphic sex chromosomes, but in *R. hastatulus*, the original Y chromosome occurring in T race is strongly heterochromatinized (it shows numerous DAPI-positive bands all over its length). Its arms were later incorporated into two neo-Y chromosomes of the NC race (Grabowska-Joachimiak et al. 2015). The recently acquired autosomal part of North Carolina Y2 chromosome did not show any cytological signs of heterochromatinization, although some evidence of genetic degeneration of this segment was provided by Hough et al. (2014). The authors investigated the molecular evolution of *R. hastatulus* sex chromosomes and showed the loss of some Y-linked genes (causing partial X chromosome hemizygosity), the ongoing diversification of sex-linked genes, and the presence of two evolutionary strata (the younger one and the older one) in sex chromosomes of the NC race.

Although cytologically distinct, the two *R. hastatulus* races hybridize in nature (Jackson and Smith 1969 in Bartkowiak 1971). However, there is no information about the meiotic chromosome configuration or mechanisms affecting viability or fertility of interracial hybrids in this species. In animals possessing sex chromosomes, one of the most common phenomena accompanying speciation is sterility and/or rarity of heterogametic sex in hybrids (Haldane’s rule, Haldane 1922). Generally, Haldane’s rule for male rarity (but not necessarily for male sterility) is strongly associated with the hemizygosity of X chromosome caused by the degeneration of Y-linked genes. The greater the Y degeneration, the greater extent of hemizygosity and opportunity for female bias among hybrids. In plants with sex chromosomes, the degeneration of Y chromosome is weaker than that in animals (Armstrong and Filatov 2008; Bergero et al. 2015), although the evolutionary trajectories of sex chromosomes are similar in the two kingdoms (Papadopulos et al. 2015). Therefore, the strongly female-biased sex ratios in hybrids are less probable. The first and so far the only example of Haldane’s rule in plants was described for *Silene* hybrids by Brothers and Delph (2010). Further studies on the hybrids between *S. latifolia* and *S. diclinis* (differing in the sex chromosome system) showed the influence of sex chromosome mismatches on extreme rarity of hybrid males in some F2 crosses (Demuth et al. 2013). The sex chromosome systems in these species (simple vs. polymorphic one) resemble those occurring in two *R. hastatulus* races, but the multiple XX/X_1_X_2_Y_1_Y_2_ system of *S. diclinis* differs from XY_1_Y_2_ occurring in the North Carolina race. Moreover, two *Silene* species showed a uniform chromosome number (2*n* = 24), in both males and females (Howell et al. 2009). It seems very interesting to establish whether the considerable chromosomal difference between two *R. hastatulus* races affects the hybrid fertility in a similar way, the more so the serious chromosome mismatches can be predicted (Table 1).

**Table 1:** Predicted male karyotypes and meiotic configurations in parental *R. hastatulus* races and their reciprocal F1 hybrids

	Male karyotype		ChN	Configuration in meiosis	Segregation pattern in meiosis
	Autosomes	Sex chromosomes			
T	8	X_T_, Y_T_	10	4_II_, X_T_-Y_T_ (5_II_)	4A + X_T_ 4A + Y_T_
NC	6	X_NC_, Y1, Y2	9	3_II_, Y1-X_NC_-Y2 (3_II_ + 1_III_)	3A + X_NC_ 3A + Y1 + Y2
NC × T	6 + 1 (A_T3_)	X_NC_, Y_T_	9	3_II_, Y_T_-X_NC_-A_T3_ (3_II_ + 1_III_)	3A + X_NC_ 3A + Y_T_ + A_T3_
T × NC	6 + 1 (A_T3_)	X_T_, Y1, Y2	10	3_II_, X_T_-Y1, Y2-A_T3_ (5_II_)	3A + X_T_ +Y2 3A + X_T_ + A_T3_ 3A + Y1 + Y2 3A + Y1 + A_T3_

*ChN* chromosome number, *T* Texas race, *NC* North Carolina race, *A*_*T3*_ the third autosome of the T race

Because in the male T × NC hybrid two separate bivalents with sex chromosomes should be formed, after the independent segregation of their elements, 50% of microspores possessing the Y1 chromosome should be devoid of the Y2 chromosome. This creates a unique opportunity to explore whether this chromosome contains vital genes, necessary for proper development/functioning of the gametophyte and/or sporophyte.

Two chromosome systems began to evolve independently in two *R. hastatulus* races about 600,000 years ago, which must have deepened the differences between them (Hough et al. 2014). If so, then the question arises, to what extent they are still compatible. For this purpose, and for the check of homology between the third chromosome of the Texas race to the translocated autosomal segments in neo-X (X_NC_) and neo-Y (Y2) chromosome in the North Carolina race, we have conducted studies on reciprocal hybrids between these races, covering the mitotic chromosomes, sex chromosome behavior in male meiosis, pollen viability, and sex ratio.

## Materials and methods

### Plant material

The investigation was carried out on specimens of *Rumex hastatulus* representing the T race, the NC race, and the F1 hybrids derived from the reciprocal crossing (T × NC) and (NC × T). All specimens were cultivated from seeds obtained from plants growing in the Department of Plant Breeding and Seed Science, University of Agriculture in Kraków. The individuals used in the current studies were grown at a temperature of 20 °C, under horticulture grow lights optimized for the flowering phase (Phytolite HPS Bloom Spectrum 400W) with a photoperiod of 12 h.

In 2011, the initial seed sample of the Texas race was received from the Royal Botanic Garden, Kew, UK, and this of the North Carolina race (collected from two populations: Marion and Gladys) was kindly provided by Professor Spencer Barrett, University of Toronto, Canada. In this study, only NC Marion plants were used.

The quantitative data on the analyzed material are presented in Table 2.

**Table 2:** Quantitative data regarding the analyzed material

	Number of plants of each form	Average number of cells/grains analyzed per plant	Number of cells/grains analyzed in total
Mitosis	10	10	400
Meiosis (DAPI)	15	30	1800
Meiosis (C-banding/DAPI)	15	10	600
Pollen stainability	15	200	12,000

### Mitotic chromosome preparation

The root tips were pretreated with a saturated solution of α-bromonaphthalene for 24 h. After fixation in 3:1 absolute alcohol:glacial acetic acid, they were hydrolyzed in 1 M HCl at 60 °C for 13 min and then squashed in 45% acetic acid. The squashes were frozen, air-dried, and conventionally stained with 0.1% aqueous solution of toluidine blue. For chromosome counting, well-spread metaphases were selected and analyzed under a Nikon Microphoto-FXA microscope equipped with a Nikon Ds-Fi1c camera and the NIS Elements software.

### Meiotic chromosome preparation

The male meiosis studies were performed on the pollen mother cells (PMCs), primarily at the stage of diakinesis and metaphase I. For this purpose, young inflorescences of male individuals were collected and fixed in a mixture of glacial acetic acid and absolute ethanol (1:3, *v*/*v*). From each inflorescence, 10 flower buds with a diameter of ca. 1 mm were selected. Subsequently, the content of pollen sacs was separated onto the slides and squashed in 45% acetic acid. After freezing in liquid nitrogen, the squashes were incubated in 96% ethanol for 15 min at 4 °C and air-dried.

The main course of meiosis in PMCs, the chromosome number, and general mode of chromosome conjugation were analyzed in preparations stained with 4′,6-diamidino-2-phenylindole (DAPI) in Vectashield (R) mounting medium (Vector Laboratories). For more precise studies of the sex chromosome configuration, the best preparations were selected and then differential staining method C-banding/DAPI was used according to Grabowska-Joachimiak et al. (2011). The procedure was slightly modified by extending the time of incubation: in 45% acetic acid to 40 min and in 2× SSC buffer to 2.5 h.

Chromosome observations were made using a Nikon Eclipse E800 microscope. Images were captured and processed with a Nikon DS-2MBWc camera and the NIS Elements software.

### Pollen stainability/fertility and sex ratio

Fresh pollen collected from several flowers was transferred onto a microscope slide and stained with 1% acetocarmine. After 30 min of staining, observations were made under a Nikon Microphoto-FXA microscope (at magnification × 40). Images were captured with a Nikon Ds-Fi1c camera and processed with the NIS Elements software. Stained (viable) and unstained (unviable) pollen grains were counted in each field of view for a total count of 200 pollen grains per slide.

All the flowering *R. hastatulus* plants were obtained from seeds harvested in the preceding year. In the case of hybrids, they were always F1 plants (grown from seeds obtained from crossing of the original chromosomal races). In total, 461 plants were sexed (at least 100 per one form).

## Results

### Chromosome complements of hybrid plants

**Fig. 2:**
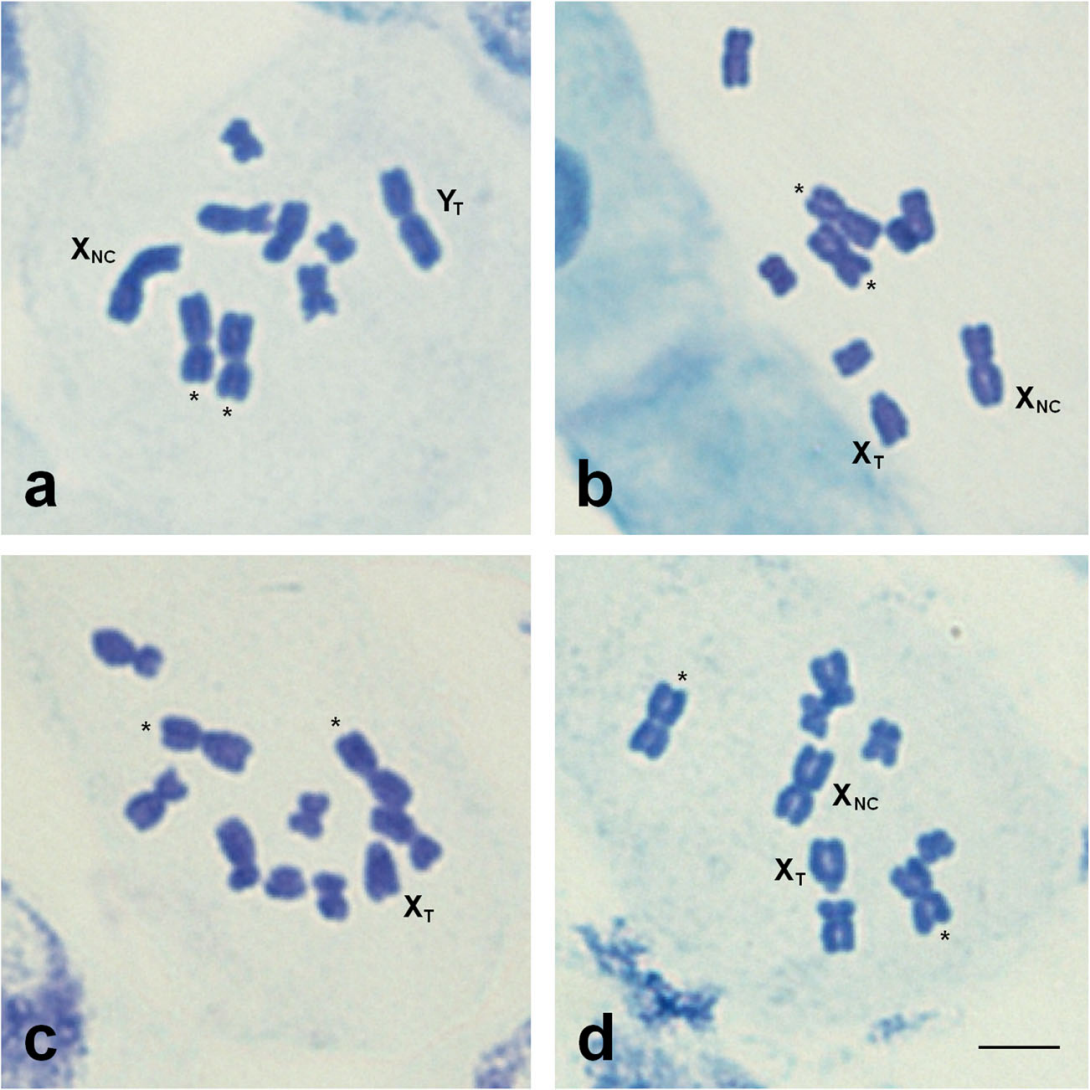
Metaphase chromosomes of interracial *R. hastatulus* hybrids. **a**, **b** NC × T; **c**, **d** T × NC. **a**, **c** Male metaphase plates. **b**, **d** Female metaphase plates. The longest autosomes are indicated by *asterisks*. Bar 5 μm

Karyotype of the NC × T hybrids (2*n* = 9) consisted of seven autosomes + two sex chromosomes: X_NC_ + Y_T_ in males and X_NC_ + X_T_ in females (Fig. 2a, b). In males, four large chromosomes were observed: the longest pair of autosomes and two sex chromosomes, from which the bigger one was the Y_T_ chromosome. Of the five remaining autosomes, two were medium-sized and three were clearly smaller (Fig. 2a). In females only, three large chromosomes were observed: the longest pair of autosomes and X_NC_ chromosome. The other medium-sized X_T_ chromosome was easy to recognize due to its specific morphology (a very small shorter arm). The remaining five autosomes were the same as in males (Fig. 2b).

The chromosome complement of the T × NC hybrids consisted of seven autosomes and sex chromosomes: X_T_ + Y_1_ + Y_2_ in males and X_NC_ + X_T_ in females. Thus, there is a difference in the chromosome number between male (2*n* = 10) and female (2*n* = 9) plants. From the four large chromosomes in males, the biggest are two metacentric autosomes (Fig. 2c). The third sex chromosome (X_T_) was easy to identify by morphology. The remaining five autosomes were the same as described in the NC × T hybrids. The female chromosome complement of this hybrid (Fig. 2d) was identical to the described above for the NC × T females.

All the analyzed F1 hybrids showed the karyotype predicted from chromosome complements of parental *R. hastatulus* races (Table 1).

### Meiotic chromosome configurations

**Fig. 3:**
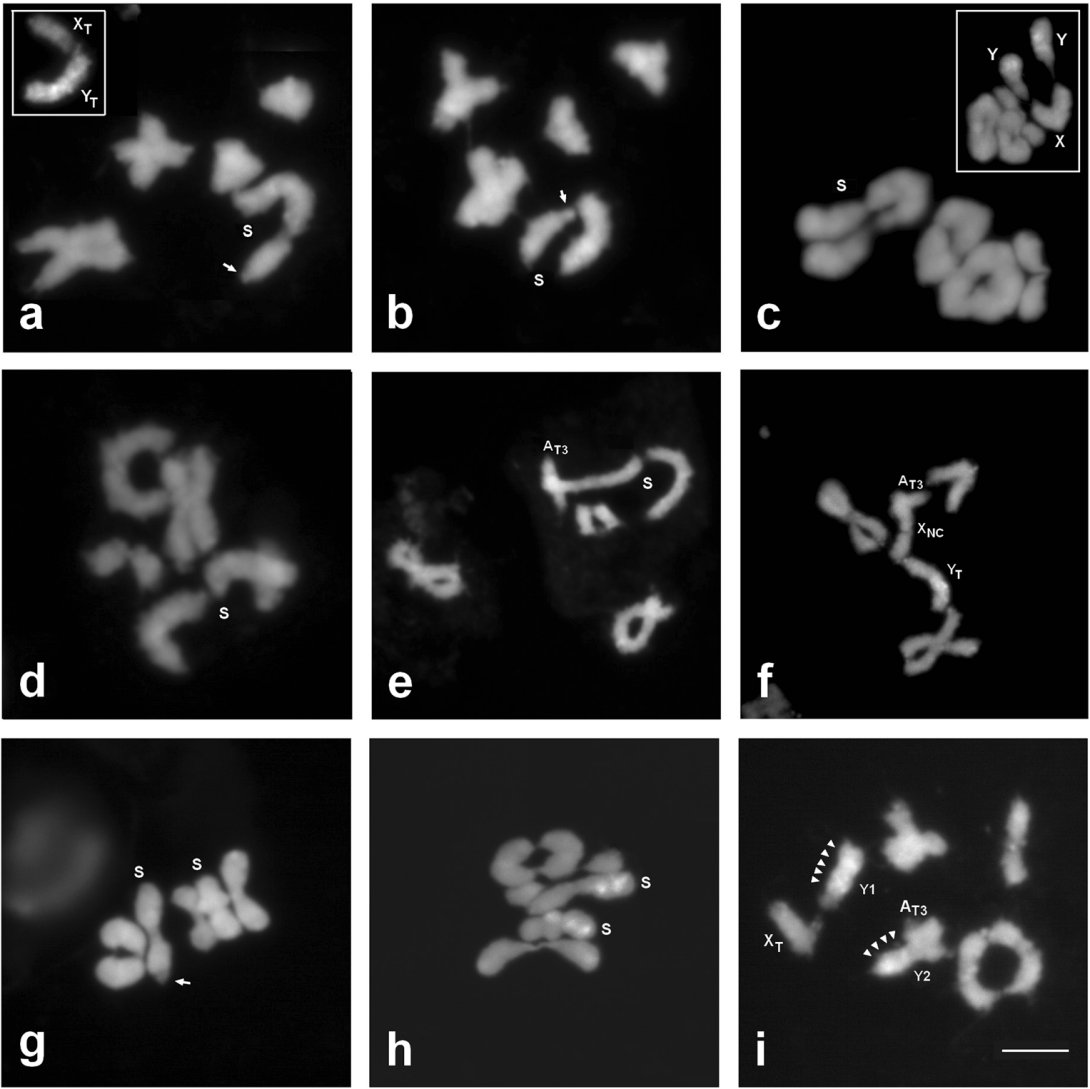
Diakinesis and metaphase I in PMCs of *R. hastatulus* after DAPI (**a**–**e**, **g**) and C-banding/DAPI staining (**a**, **c** frames, **f**, **h**, **i**). **a**, **b** Texas race. **c** North Carolina race. **d**–**f** NC × T hybrid. **g**–**i** T × NC hybrid. S, sex chromosome association. *Arrows* point to the short arm of X_T_ chromosome. *Arrowheads* indicate C-banding/DAPI-positive heterochromatin. Bar 5 μm

The course of male meiosis in parental forms and their hybrids was regular. No laggards, chromosome bridges, or micronuclei were observed in the analyzed cells. In the first meiotic division of PMCs, chromosomes of the T race and the T × NC hybrids formed five bivalents (Fig. 3a, b, g–i), but chromosomes of the NC race and the NC × T hybrids formed three bivalents and one trivalent (Fig. 3c–f).

The sex chromosomes in the T race formed a bivalent, and in the NC race, a trivalent. In the T race, sex chromosomes are well distinguishable in DAPI-stained preparations (X is smaller than Y), but in the NC, they are not. Interestingly, the strongly heterobrachial X chromosome of the T race was differently oriented in relation to Y chromosome—in some bivalents, it was joined with the male sex chromosome by its short arm, in others by the long arm (Fig. 3a, b). It suggests the occurrence of pseudoautosomal regions on both ends of X_T_ chromosome. C-banding/DAPI enables reliable identification of heterochromatinized Y chromosomes in meiosis of the two *R. hastatulus* races (frames in Fig. 3a, c); the predicted Y1-X_NC_-Y2 chromosome orientation in sex trivalent of the North Carolina race was definitely confirmed.

The trivalent in the NC × T males consisted of two large sex chromosomes (Y_T_ and X_NC_) and the small A_T3_ autosome (Fig. 3d). The participation of A_T3_ in this association was particularly well visible at diakinesis where chromosomes were less condensed (Fig. 3e). However, in DAPI-stained preparations, the predicted order of chromosomes in this chromosome association (Y_T_-X_NC_-A_T3_) was difficult to confirm because of the similar length of sex chromosomes. It turned out to be possible in C-banding/DAPI preparations in which Y_T_ showed brightly stained heterochromatin (Fig. 3f).

There were two bivalents with sex chromosomes in the T × NC males (Y1-X_T_ and Y2-A_T3_). Both were heteromorphic, thus well identifiable in DAPI-stained preparations. In all observed cases, X_T_ chromosome in one of such bivalents was joined with Y chromosome by its long arm (Fig. 3g). The identification of Y chromosomes within the first metaphase bivalents was possible after C-banding/DAPI, but they were indistinguishable from each other at this stage (Fig. 3h). At diakinesis, in a less condensed state, Y2 chromosome showed, in contrast to Y1, a euchromatic arm (Fig. 3i). In all the analyzed preparations, it was joined by this arm with the small A_T3_ autosome (Y2-A_T3_ bivalent). Largely heterochromatic Y1 chromosome was connected with X_T_ chromosome by a small euchromatic (pseudoautosomal) segment (Y1-X_T_ bivalent) (Fig. 3i).

The observed meiotic configurations were fully consistent with the predictions based on the translocation hypothesis. They finally confirmed the autosomal (A_T3_) origin of the segments translocated on the North Carolina neo-sex chromosomes and their role in the fixation of the polymorphic sex chromosome system in this race.

### Morphology and stainability of pollen grains

To assess the fertility of hybrid males, we compared the morphology and stainability of their pollen grains with the morphology and stainability of pollen grains in the parental forms.

The majority of pollen grains produced by the Texas race, the North Carolina race, and NC × T hybrids were uniformly sized and showed no visible signs of degeneration. They were regular in shape and abundantly filled with starch grains. The frequency of abnormal grains (dwarf, empty, non-regularly shaped) was 1.52% in T, 2.21% in NC, and 2.55% in NC × T. In pollen sacks of T × NC hybrids, much more abnormal pollen grains were observed (~ 30%).

**Fig. 4:**
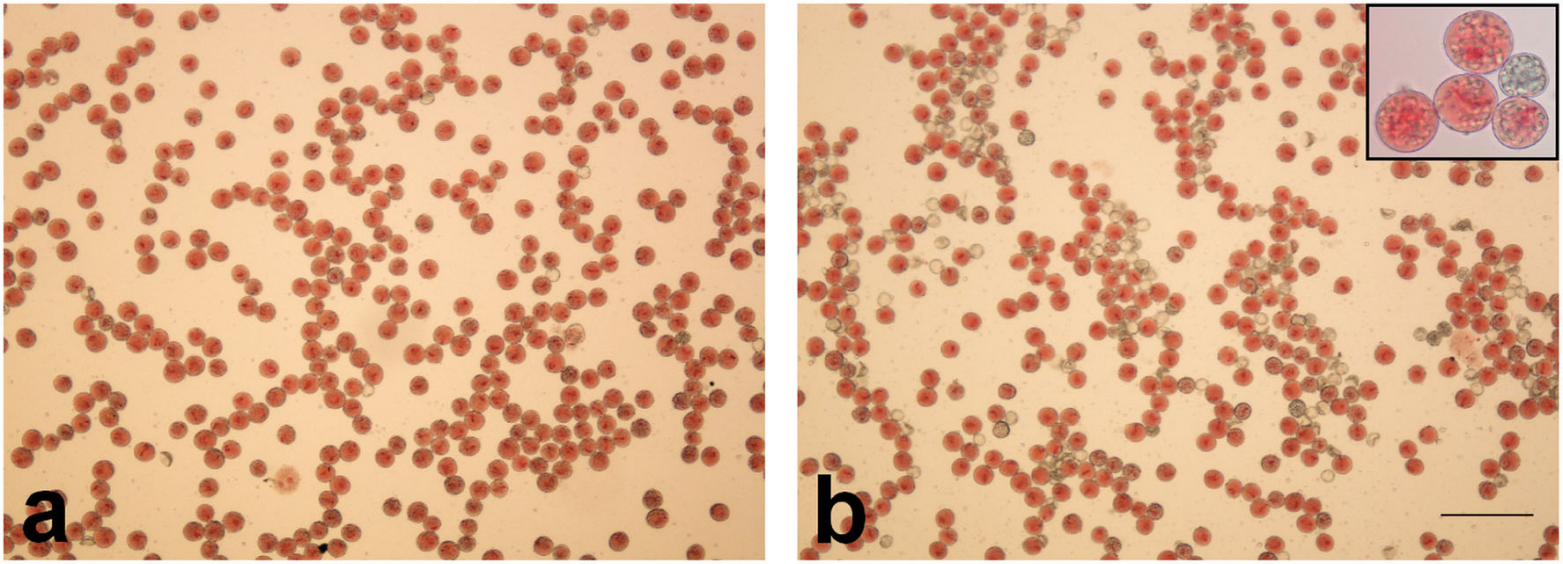
Pollen grains of *R. hastatulus* hybrids stained with acetocarmine. **a** NC × T and **b** T × NC. Note stainable and non-stainable pollen grains filled with starch (**b**, frame). Bar 50 μm

The acetocarmine test showed that in all of the analyzed forms, the vast majority of normally developed grains were colored red, and that the non-typical grains were predominantly unstained, although a few of them were abundantly filled with starch grains (Fig. 4).

The percentage of acetocarmine-stained pollen grains in Texas, North Carolina, and NC × T males was similar (98.37%, 97.87%, and 97.2%), but in T × NC males, it was dramatically reduced (69.8%) (Table 3).

**Table 3:** Frequency of stainable (S) and non-stainable (N) pollen grains in *R. hastatulus* males

Texas		North Carolina		NC × T		T × NC	
S	N	S	N	S	N	S	N
2951	49	2936	64	2916	84	2094	906

The difference in frequency of stainable and non-stainable pollen grains in the two chromosomal *R. hastatulus* races turned out non-significant, like between the NC race and NC × T hybrid. The difference between T × NC hybrid and all other forms was highly significant (*P* < 0.0001) (Table 4).

**Table 4:** Results of pairwise Pearson’s chi-square test (*P* values) for proportion of stainable and non-stainable pollen grains in *R. hastatulus* males

	T	NC	NC × T	T × NC
T	–			
NC	0.1542	–		
NC × T	0.0021	0.0960	–	
T × NC	< 0.0001	< 0.0001	< 0.0001	–

### Sex ratios

The expected 1:1 sex ratio was observed only in NC × T hybrid. Three other forms showed female-biased sex ratios. In the parental forms, the male-to-female ratio (calculated as 1:F/M) deviates more from the equality in the North Carolina race (1:1.46) than in the Texas race (1:1.05), where it is very close to an ideal balance. In T × NC hybrid, sex ratio is strongly female-biased (1:1.72). Only in this form the numerical predominance of female plants is statistically significant (Table 5).

**Table 5:** Proportion of males to females in the analyzed *R. hastatulus* forms

	Texas		North Carolina		NC × T		T × NC	
	M	F	M	F	M	F	M	F
PR	80	84	46	67	59	59	39	67
%	48.78	51.22	40.71	59.29	50	50	36.79	63.21
*P*	0.818		0.060		1.000		0.009*	

*PR* frequency of male and female plants; *P* binominal probability (two tail), approximation via normal

*Highly significant (balanced 1:1 sex ratio expected)

### Discussion

The final confirmation of the translocation hypothesis (Smith 1964; Grabowska-Joachimiak et al. 2015) required the analysis of the conjugation pattern in hybrid plants possessing the original A_T3_ chromosome(s) and neo-sex chromosomes (X_NC_ and/or Y2) in the karyotype. In this work, we obtained such hybrids and analyzed meiotic chromosome configurations in their pollen sacks. It was shown that both X_NC_ and Y2 chromosomes possess a terminally located segment homologous to the third autosome of the Texas race. Thus, the translocation hypothesis has been fully confirmed. Thanks to the C-banding/DAPI staining, it was also possible to identify hardly distinguishable Y1 and Y2 chromosomes within meiotic associations of the NC race and T × NC hybrid. The analysis of meiotic figures in the T and NC races confirmed previous reports (Smith 1955, 1963, 1964; Bartkowiak 1971). The analysis of the chromosome conjugation in interracial hybrids was made for the first time.

**Fig. 5:**
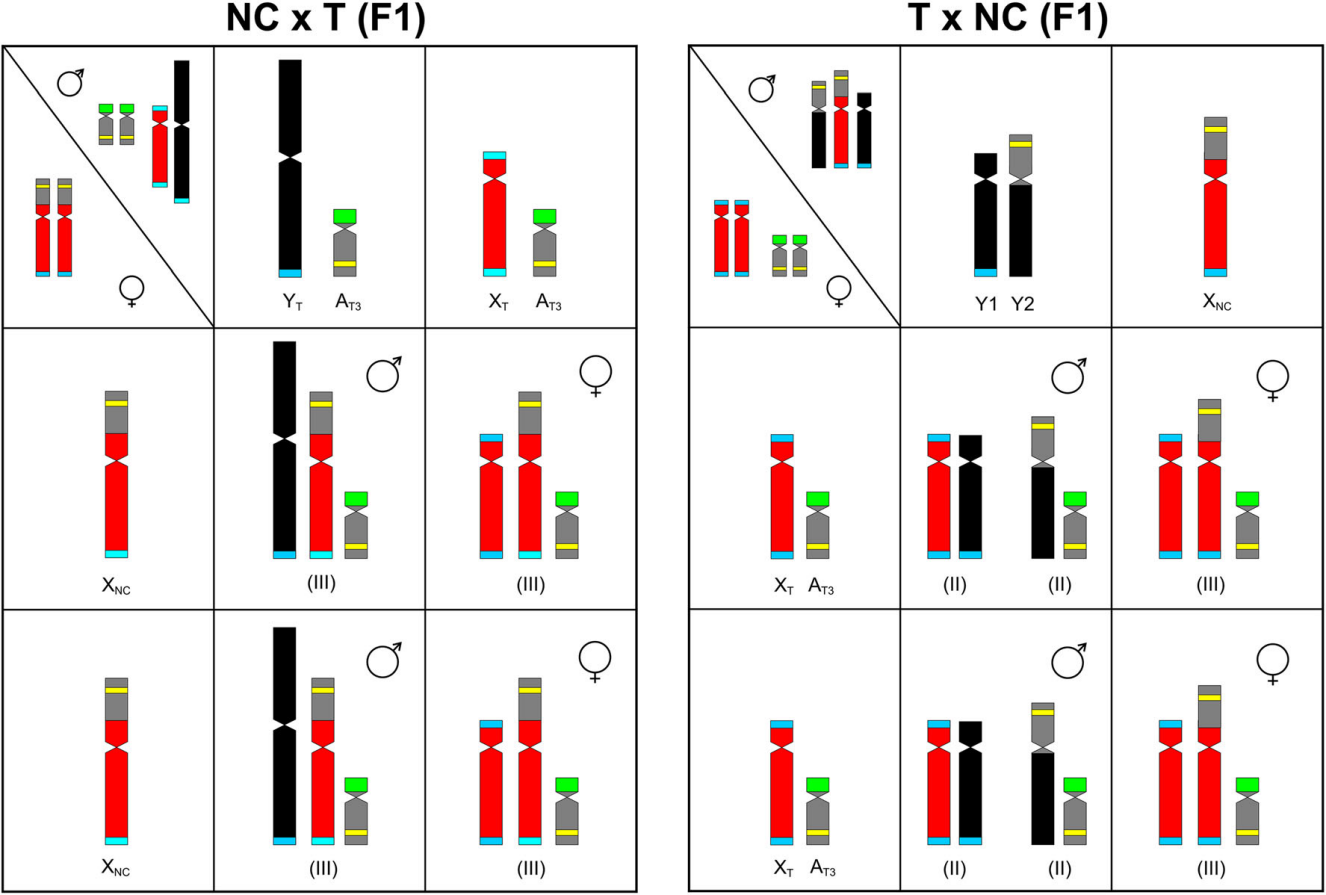
Sex and A_T3_ chromosome configurations in parental races and F1 hybrids of *R. hastatulus*: NC × T and T × NC. (II)—bivalent, (III)—trivalent

After 600,000 years of independent evolution, the autosomes and sex chromosomes of two *R. hastatulus* races seem to be still compatible. In both NC × T hybrid, possessing Y_T_ and X_NC_ chromosomes (Fig. 5a), and T × NC hybrid, possessing X_T_ and Y1+Y2 chromosomes (Fig. 5b), meiosis proceeds without disturbances. The F1 females showed the same karyotype and chromosome number (2*n* = 9) in both hybrids, whereas males differed in this feature (2*n* = 9 vs. 2*n* = 10). The 2*n* = 9 chromosome number was quite new for the female sex in *R. hastatulus*, and it has never been found in the original races.

**Fig. 6:**
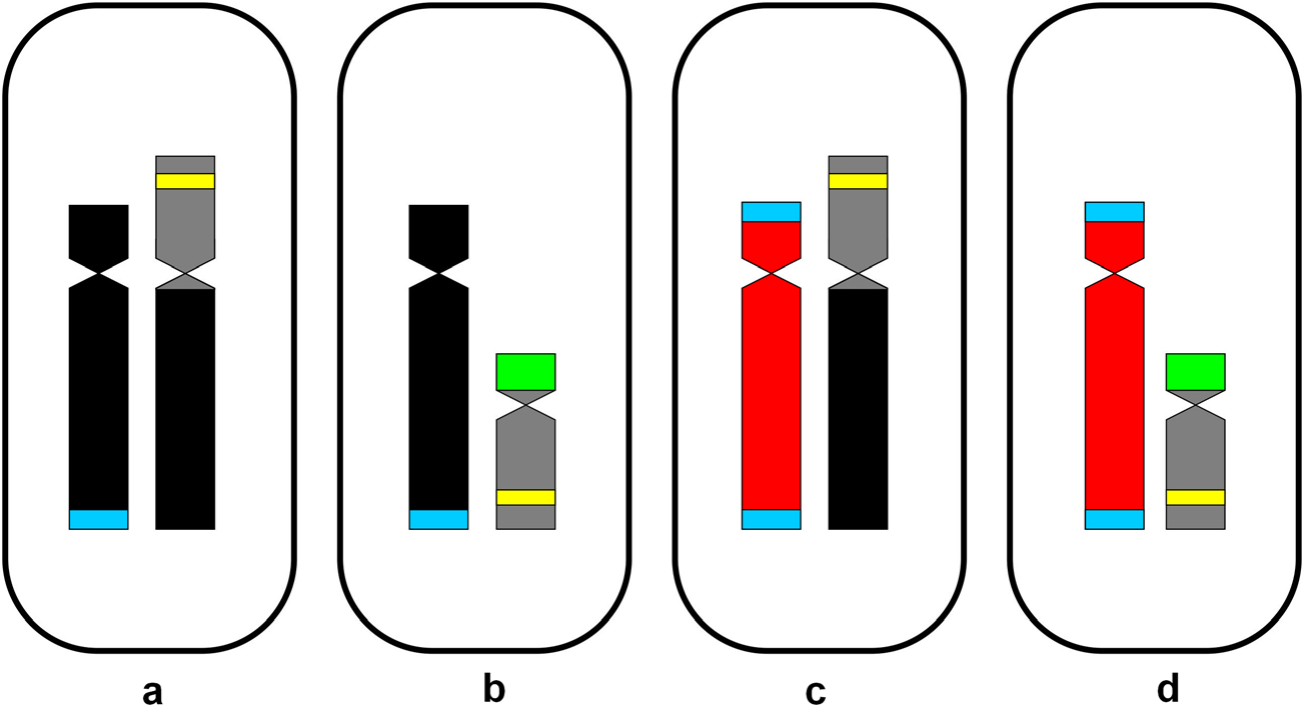
**a**–**d** Four types of microspores produced by T × NC males, **b**—probably inviable

The result of acetocarmine staining and morphological inspection of pollen grains showed that the pollen viability in male NC × T hybrid was high (~ 97%), quite similar to parental lines. It turned out, however, that in T × NC hybrid, the pollen viability was definitely reduced. The reason for such reduction has not been thoroughly investigated, but most probably it was associated with the presence of two sex bivalents (X_T_ + Y1 and Y2 + A_T3_) in males of this form. Due to the independent chromosome segregation, four different haploid chromosome sets can be produced during meiosis (Table 1). Three quarters of the resulting microspores inherit the X chromosome or two neo-Y chromosomes (an equivalent of Y_T_ chromosome), but one quarter is deprived of one of the Y chromosomes (has only Y1 chromosome) (Fig. 6b). It causes, in turn, formation of unviable gametophytes, because the presence of both Y chromosomes (or the X chromosome) seems to be essential for normal development of pollen in *Rumex* with the polymorphic XX/XY1Y2 sex chromosome system (Żuk 1970a, 1970b). The presence of genetic elements controlling male fertility in Y chromosomes is a common phenomenon. Even in highly heterochromatinized animal Y chromosomes, genes responsible for this function were preserved for a long time (Steinemann and Steinemann 2000 and references therein).

The XY chromosomes contain certain genes required for cell functioning and plant development. The loss of recombination drives degeneration of some “sporophytic” genes in the Y chromosome, but all vital genes necessary in male gametophyte have to be preserved (Chibalina and Filatov 2011; Charlesworth 2015; Crowson et al. 2017). For this reason, the genetic information contained in the intact Y chromosome(s) is sufficient for the proper male gametophyte development but insufficient in the sporophyte. Thus, in plants with heteromorphic sex chromosomes, the sporophytes lacking X chromosomes (e.g., 2A + YY) are not observed (Westergaard 1958). It is also true for *R. hastatulus*, where the failure of the expected 2A + Y1Y1Y2Y2 genotypes among the progeny of the self-pollinated 2A + XY1Y2 andromonoecious intersexes was evidenced (Smith 1963). On the other hand, the plant Y chromosome(s) seems to be not superfluous in the sporophyte development. To our knowledge, 2A + X plants have never been observed so far in species with sex chromosomes. This may be due to the lack or low level of dosage compensation in plants (Muyle et al. 2017). It appears that in the diploid phase of life cycle, at least one X chromosome plus any other sex chromosome is required. This suggests that some dosage-sensitive genes expressed in the sporophyte are preserved in Y chromosomes. It may be conditioned by their necessity in the gametophyte or by the inability to develop an efficient dosage compensation mechanism. The research carried out on this subject so far has not yielded conclusive results (Muyle et al. 2017).

The frequency of stainable pollen grains in 15 analyzed T × NC hybrid males (69.8%, on average) was lower than predicted from the frequency of the defective microspores (75%), and this difference showed to be statistically significant (*P* = 0.0285, *N* = 15, SD = 8.25; one sample *t* test). However, it should be taken into account that the non-stainable pollen was also observed in three other forms (with average frequency of 2.2%). So the predicted frequency of non-stainable pollen grains in T × NC hybrid should be lowered by this value. Having considered this correction, the mentioned difference turned out statistically insignificant (*P* = 0.1808). This indicates the production of Y-defective microspores as the leading cause of an increase of pollen inviability in T × NC males.

**Fig. 7:**
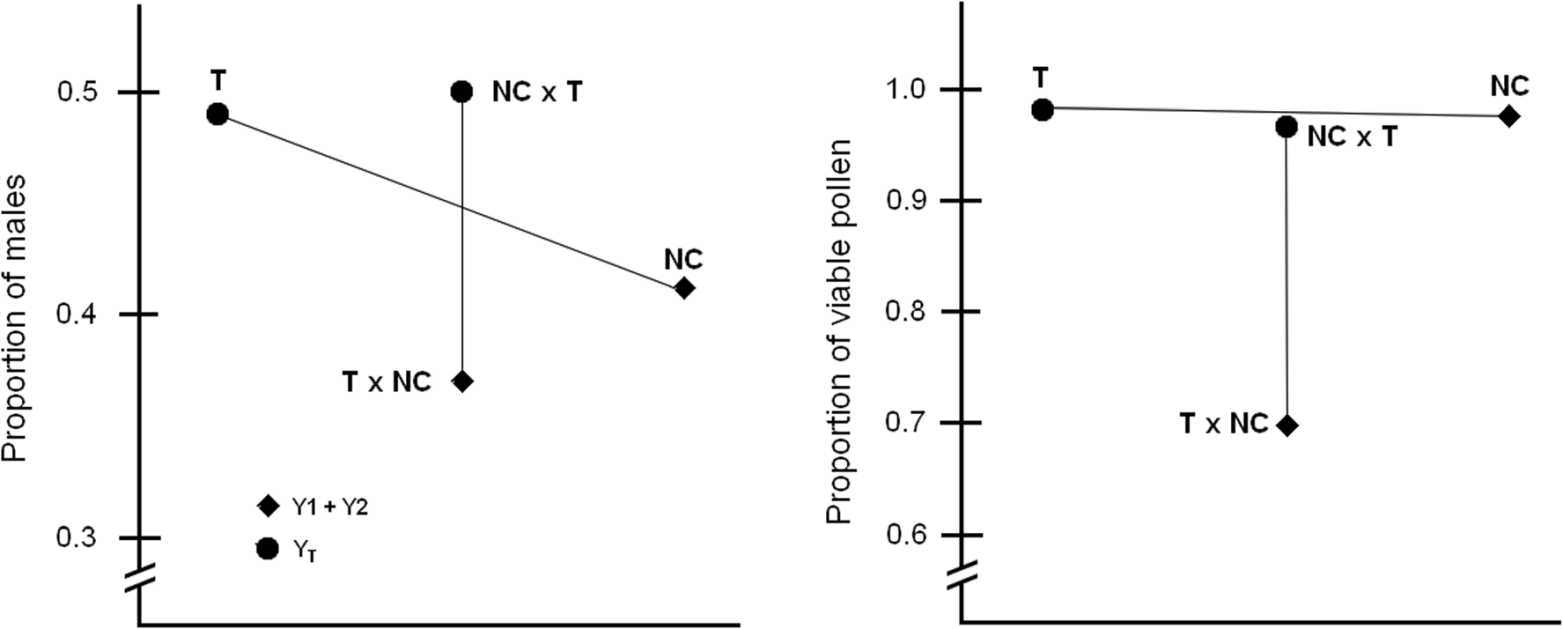
Proportion of males (males/total sample) and viable pollen (viable/total sample) in four *R. hastatulus* forms differing in sex chromosome composition

Among the T × NC hybrids, a strong quantitative advantage of females was observed (1.72:1). Female predominance was also observed in the NC race, but to a lesser extent (1.46:1). In statistical terms, however, the sex ratio difference between NC race and T race was not significant (*P* = 0.1846, *N* = 277; Pearson’s chi-square test for proportion). Interestingly, males of female-biased forms (NC and T × NC) are equipped with neo-Y chromosomes (Y1 and Y2) in contrast to the T race and NC × T hybrid (possessing Y_T_ chromosome), in which the sex ratio was close to equality (Fig. 7). Confirmation of the discovered relationships requires further studies on more extensive material.

Our results concerning the difference in male-to-female proportion in progeny of parent races do not agree with the data from 46 natural populations of *R. hastatulus* (Pickup and Barrett 2013), where both Texas and North Carolina showed mostly female-biased sex ratios. However, these results are incomparable because the plants we examined germinated, developed, and flowered in uniform experimental conditions.

Although many different mechanisms leading to the female-biased sex ratios in dioecious plants were proposed (Barrett et al. 2010), the majority of them do not apply to the newly formed interspecific or interracial hybrids. In hybrids between forms possessing heteromorphic sex chromosomes, the absence or rarity of males (Haldane’s rule for male rarity) is most likely caused by the incompatibilities involving heterosomes. In animals, this phenomenon is strongly conditioned by the X chromosome hemizygosity caused by the extensive Y chromosome degeneration (Brothers and Delph 2010 and references therein). In rare plants with heteromorphic sex chromosomes, the degree of Y degeneration and hence X hemizygosity is much smaller (Chibalina and Filatov 2011; Hough et al. 2014; Bergero et al. 2015; Muyle et al. 2017), so the male underrepresentation among hybrids (if observed) must be achieved in a different way. Indeed, in *Silene* where Haldane’s rule (for both male rarity and fertility) was firstly reported, the low frequency of males in part of hybrids was most likely caused by the zygotic aneuploidy involving neo-sex chromosomes (Demuth et al. 2013), although some contribution of a small amount of Y degeneration cannot be ruled out. This aneuploidy was a consequence of aberrant segregation of sex chromosomes in some paternal plants.

*Rumex hastatulus* is the second plant in which Haldane’s rule for male rarity and male fertility was evidenced. Like in *Silene*, the deficiency of males was observed in hybrids possessing heterosomes from two different sex chromosome systems (simple vs. polymorphic). Moreover, both plants showed asymmetry in Haldane’s rule in male rarity—the frequency of male plants was significantly reduced only in the hybrid inheriting neo-sex chromosomes from their father. In the hybrid resulted from the opposite cross, sex ratio was unaffected and similar to the one observed in the pure parents. Generally, the cross asymmetry in Haldane’s rule (named “Darwin’s corollary”) is widespread in animals but it is not dependent on the co-occurrence of heterosomes from two different sex chromosome systems in a specific configuration (Turelli and Moyle 2007 and references therein).

There are, however, some important differences between mechanisms governing Haldane’s rule in *Rumex* and *Silene* hybrids. In *Rumex*, Haldane’s rule was highly asymmetrical not only for male rarity but also for male infertility: fertility of NC × T hybrid was close to the one observed in parents, while the fertility of T × NC hybrid was greatly reduced (Fig. 7). This contrasts with the marked reduction of male fertility observed in *Silene* hybrids, regardless of the direction of the cross (Brothers and Delph 2010). The reason for this disparity may be the multifaced incompatibility of nuclear and organellar genomes coming from different species and the complex genetic system controlling sex ratio in *Silene* (Taylor 1994a, 1994b).

The conducted research also yielded some progress in determination of the location of genetic factors responsible for proper pollen development in *R. hastatulus*. Most probably, they are located on the Y2 chromosome of North Carolina race, because the microspores lacking this chromosome are probably not able to develop into viable pollen. According to the translocation hypothesis, the non-recombining part of this chromosome was inherited from the ancestral Y chromosome (it is an equivalent of p arm of Y_T_ chromosome) (Fig. 1). Because Y chromosomes of *R. hastatulus* are not neutral in gender determination (Smith 1963; Bartkowiak 1971), they should also contain genetic material responsible for the expression of maleness. It will be possible to localize it within *R. hastatulus* Y chromosome(s) through analysis of the back-cross hybrids between T and T × NC. Due to the presence of the X_T_ + Y2 pollen grains (Fig. 6c), one third of such hybrids should have two X_T_ chromosomes plus Y2 chromosome. If the Y2 chromosome contains the male-promoting region, intersexual plants should be produced. Otherwise, this region should be assigned to Y1 chromosome (which is the equivalent of the q arm of Y_T_ chromosome).
